# Habit-formation strategies enhance long-term outcomes of a self-compassionate touch micropractice: a randomized controlled trial

**DOI:** 10.1038/s41598-026-63457-4

**Published:** 2026-09-15

**Authors:** Eli S. Susman, Serena Chen, Dacher Keltner, Allison G. Harvey

**Affiliations:** Department of Psychology, University of California, Berkeley, 2121 Berkeley Way, Berkeley, CA 94720 USA

**Keywords:** Diseases, Health care, Psychology, Psychology

## Abstract

This preregistered study tested a 15-minute, self-guided, online, single-session intervention designed to promote habit formation of self-compassionate touch micropractice (≤ 20 s/day). A U.S. national adult sample (*N* = 497) was randomized to self-compassionate touch with habit formation tools (SCT+HABITS) or self-compassionate touch alone (SCT)—and assessed at baseline, month-3, and month-6. Relative to SCT, SCT+HABITS showed greater baseline-to-3-month and baseline-to-6-month increases in practice frequency, practice automaticity (habit formation), self-compassion, and self-compassion automaticity, as well as greater baseline-to-6-month reductions in symptoms of psychopathology (mental health symptoms). Baseline-to-3-month increases in practice automaticity mediated baseline-to-6-month effects, accounting for approximately 40–83% of the total group effects. Both interventions demonstrated high acceptability. Their outcomes did not differ by racialized identity. Findings highlight the role of habit formation in promoting positive outcomes of self-compassionate touch micropractice, and perhaps well-being practices more broadly. The scalability and adaptability of SCT+HABITS highlight a promising model for advancing efficient, effective, user-friendly interventions.

**Trial registration** ClinicalTrials.gov Identifier: NCT05866718, registered 23/05/2023.

**Supplementary Information:**

The online version contains supplementary material available at https://doi.org/10.1038/s41598-026-63457-4.

## Introduction

Up to 40% of premature deaths and health problems are preventable through the habitual practice of health behaviors^1,2^. Despite the importance of healthy habits^1,2^, efforts to build them more often fail than flourish^3,4^.

Well-being practices are a category of health behaviors defined here as “practical methods to bring about a state of enduring well-being or inner flourishing […] by voluntarily cultivating healthy habits of mind (p. 121)”^5^. Emotional well-being, a multidimensional construct encompassing emotional states, life satisfaction, purpose, and positive functioning^6^, is a core target of such practices. Well-being practices can promote a range of psychological benefits^7,8^, yet they often fail to endure as daily habits^9,10^. Because practice frequency is a key contributor to the benefits of well-being practices^10–13^, this study seeks to test the hypothesis that adding tools to promote habit formation will improve outcomes of well-being interventions by promoting practice habits^14^.

This study focused on self-compassion practice. This subset of well-being practices aims to cultivate the five core elements of self-compassion: acknowledging suffering, seeing suffering as a universal human experience, emotionally connecting with the suffering, being able to tolerate uncomfortable feelings, and being motivated to alleviate one’s suffering^15,16^.

Self-compassion practice has a range of documented benefits, including reducing stress and symptoms of psychopathology^7,10^. Yet the most widely used interventions for promoting self-compassion demand significant time commitments. A Mindful Self-Compassion program consists of eight, two- or two-and-a-half-hour sessions and a half-day retreat^17^. Research on Compassion-Focused Therapy suggests that at least twelve one-hour sessions may be needed to significantly reduce symptoms of psychopathology across clinical populations^18,19^. Self-compassion interventions typically span 1 to 20 contact hours, coupled with 2.5 to 40 h of independent practice after learning the intervention^7^. Such time commitments render them inaccessible to a large portion of the population, as studies consistently identify lack of time as the primary barrier to engaging in well-being interventions^7,20–22^, even when practice sessions are only 5 to 15 min in duration^20,22^. Thus, although brief (i.e., 5- to 15-minute) self-compassion practices have shown promise^23–25^, engagement with these interventions remains a challenge^20,22,23^, highlighting the need for still-lower-burden approaches that can be practiced consistently amid the time constraints of daily life.

Micropractice offers a promising approach to addressing this barrier^10^. Micropractice consists of ultra-brief (i.e., ≤ 30-second) training sessions distilled from the most potent elements of well-being practices and can be accessed with minimal burden. This approach not only lowers the barriers to entry but also reduces the time and commitment needed to pursue meaningful gains.

Self-compassionate touch is one promising micropractice, involving self-touch intended to evoke feelings of self-compassion, while focusing on feelings of warmth and/or self-compassionate thoughts^10,26^. Touch engages an evolutionarily conserved emotion-regulation system via C-tactile afferents that support parasympathetic regulation and reduced stress reactivity^27^. Consistent with this, in prior work, one month of daily practice increased self-compassion and reduced stress and symptoms of psychopathology relative to an active control, with effects comparable to more time-intensive well-being interventions^10^. Yet, substantial room for improvement remains, as these benefits were only observed in the 38% who practiced daily, and more frequent practice was associated with stronger effects^10^. In essence, cultivating a daily habit of self-compassionate touch may be crucial to deriving benefits.

Despite the broad application of habit formation interventions, none have specifically been tested for building the habit of self-compassion practice. This is a significant gap, as habit formation interventions have yet to demonstrate significant success in increasing the frequency or automaticity of well-being practices^28,29^. Given the difficulty many encounter in developing a daily habit of well-being practices broadly and self-compassionate touch specifically, integrating evidence-based habit formation strategies into a self-compassionate touch intervention is proposed as a promising approach to promoting practice and improving outcomes^10,14,30,31^, and is the focus of this study. This approach extends an ongoing study conducted in a different population and is consistent with recommendations to integrate habit-formation principles into evidence-based interventions^14,30,31^.

Unlike intentional behaviors, which are consciously controlled, habits are characterized by automaticity, formed through repeated (e.g., daily) practice in a consistent context until the behavior becomes automatically triggered by that context^32^. Two types of automaticity are measured in this study: practice automaticity, reflecting the extent to which individuals initiate daily self-compassionate touch with minimal conscious effort, and self-compassion automaticity, reflecting the spontaneous expression of self-compassion in daily life. Automaticity is crucial because when habits compete with intentions, habits most often prevail^32^. Applying these concepts to self-compassionate touch, repeatedly pairing thoughts that evoke feelings of unworthiness with self-compassionate responding may increase the likelihood of self-compassion in similar real-life situations. So, cultivating habits could enhance the practice of self-compassionate touch (via practice automaticity) and the persistence of self-compassion in daily life (via self-compassion automaticity)^32^.

This study is the first to evaluate the potential of a single-session self-guided online intervention for habit formation. These interventions warrant investigation given widespread preferences for independent problem-solving^33^, growing reliance on digital platforms for emotional support^34^, their scalability, and the modal pattern of attending only one session^35^. However, they may be less effective than approaches involving human contact^9^ and may pose challenges for individuals new to well-being practices^9^. These considerations underscore the need for a tailored approach that draws on habit formation science.

This study tested whether improving practice automaticity of self-compassionate touch through a single-session self-guided online habit formation intervention improves outcomes. Adults (*n* = 497) recruited across the United States via social media were randomly assigned to either the self-compassionate touch intervention alone (“SCT”) or the self-compassionate touch intervention with habit formation tools (“SCT+HABITS”) and completed assessments at baseline, 3 months, and 6 months.

We report on pre-registered analyses for three confirmatory aims hypothesizing SCT+HABITS would be superior to SCT, and two exploratory aims. Aim 1 was to test whether SCT+HABITS, relative to SCT, produced greater increases in practice frequency and practice automaticity from baseline to 3- and 6-month follow-up. Aim 2 was to evaluate whether SCT+HABITS, compared to SCT, showed greater increases in self-compassion and self-compassion automaticity and reductions in stress and symptoms of psychopathology from baseline to 3- and 6-month follow-up. Aim 3 was to test whether greater increases in practice automaticity from baseline to 3 months mediated the association between condition (SCT+HABITS vs. SCT) and Aim 2 outcomes from baseline to 6 months.

Exploratory Aim 1 probed the acceptability of SCT+HABITS and SCT. Exploratory Aim 2 evaluated whether the predicted effects from Aims 1 and 2 differed between participants identifying as non-Hispanic white and those identifying as racialized.

## Results

### Participant characteristics and preliminary analyses

**Fig. 1:**
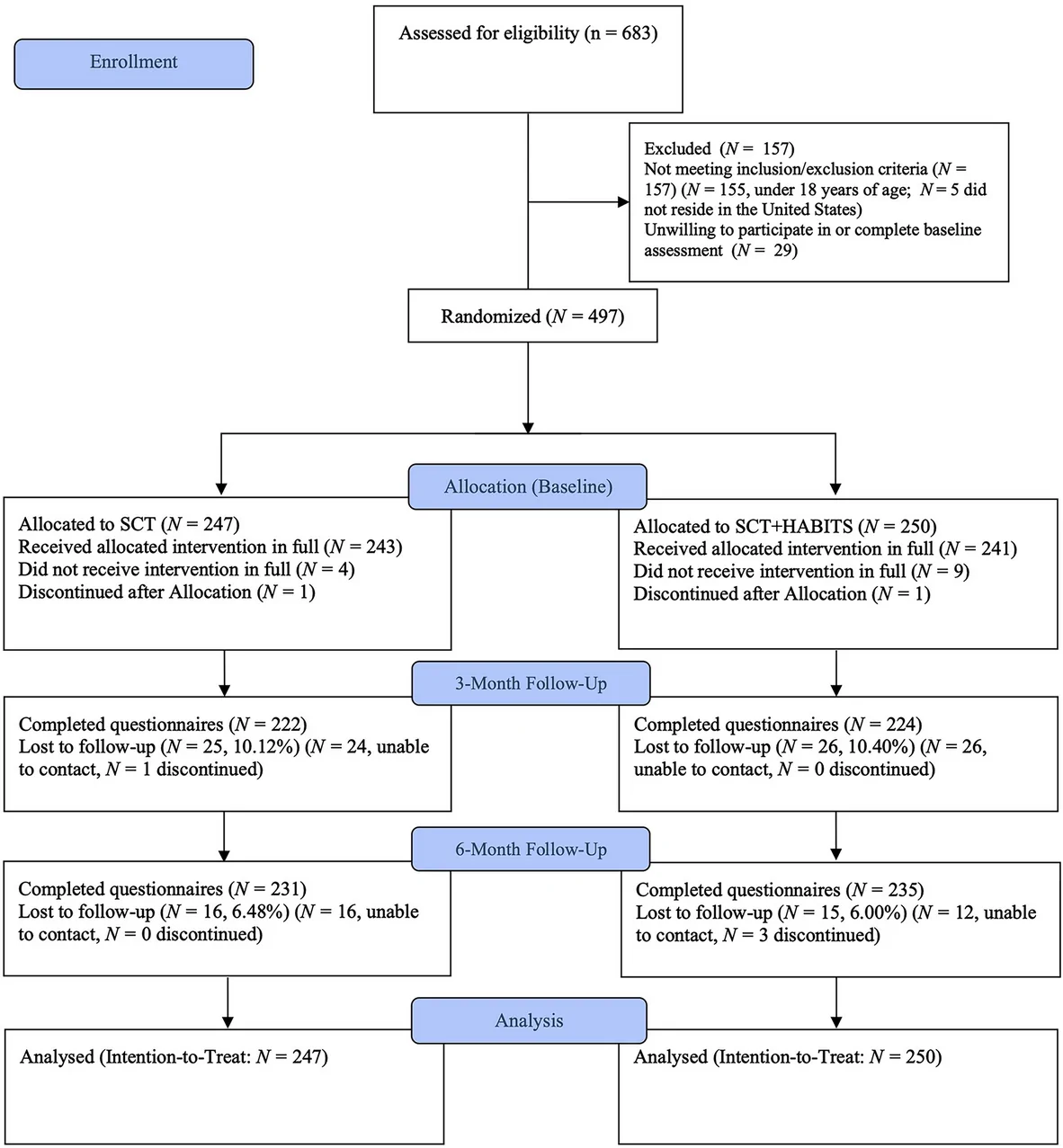
Participant flow through the study. CONSORT diagram of participant flow through eligibility screening, randomization and assessment completion.

Table 1 presents demographic variables and participant characteristics. Table 2 presents the descriptive statistics of all outcome variables. The attrition rate was 10.26% (51 participants) at 3-month follow-up and 6.24% (31 participants) at 6-month follow-up. Figure 1 presents a CONSORT diagram that details the attrition rates for each group at each timepoint. The proportion of participants who did not receive the allocated intervention in full did not significantly differ between SCT and SCT+HABITS (χ^2^ = 1.91, df = 1, *p* = .167). Relative to completers, non-completers were not significantly different in sex assigned at birth at 3-month follow-up (χ^2^ = 1.37, df = 1, *p* = .242) and 6-month follow-up (χ^2^ = 2.45, df = 1, *p* = .117), nor treatment condition at 3-month follow-up (χ^2^ = 0.01, df = 1, *p* = .919) and 6-month follow-up (χ^2^ = 0.05, df = 1, *p* = .826). At 3-month follow-up, completers were 4.24 years younger at pre-treatment than non-completers (*t* = 2.18, df = 63.81, *p* = .033). At 6-month follow-up, completers were not significantly different in age relative to non-completers (*t* = 0.75, df = 34.13, *p* = .457).

**Table 1:** Demographic variables and participant characteristics.

Characteristic	Whole sample (*N* = 497)		SCT+HABITS (*N* = 250)		SCT (*N* = 247)	
	*N*	%	*N*	%	*N*	%
Sex						
Female	260	52.31	120	48.00	140	56.68
Male	232	46.68	126	50.40	106	42.91
Missing	5	1.01	4	1.60	1	0.40
Gender						
Female	236	47.48	106	42.40	130	52.63
Male	238	47.89	128	51.20	110	44.53
Non-Binary	25	5.03	16	6.40	9	3.64
Missing	4	0.80	3	1.20	1	0.40
Not listed	2	0.40	1	0.40	1	0.40
Race/Ethnicity						
American Indian or Alaska Native	16	3.22	8	3.20	8	3.24
Asian	55	11.07	20	8.00	35	14.17
Black	24	4.83	14	5.60	10	4.05
Hispanic/Latinx	47	9.46	21	8.40	26	10.53
Native Hawaiian or other Pacific Islander	2	0.40	0	0.00	2	0.81
White	389	78.27	201	80.40	188	76.11
Multiracial	42	8.45	19	7.60	23	9.31
Not listed	3	0.60	1	0.40	2	0.81
Missing	4	0.80	3	1.20	1	0.40
Non-Hispanic White	354	71.23	184	73.60	170	68.83
Racialized	139	27.97	63	25.20	76	30.77
Relationship status						
Single	157	31.59	86	34.40	71	28.74
Committed relationship	83	16.70	36	14.40	47	19.03
Separated/Divorced/ Widowed	50	10.06	23	9.20	27	10.93
Married	204	41.05	103	41.20	101	40.89
Missing	3	0.60	2	0.80	1	0.40
Education						
Without high school diploma	1	0.20	0	0.00	1	0.40
High school graduate	51	10.26	24	9.60	27	10.93
Some college education	31	6.24	15	6.00	16	6.48
Degree from 4-year college or more	413	83.10	211	84.40	202	81.78
Missing	1	0.20	0	0.00	1	0.40
Employment						
Full time	287	57.75	150	60.00	137	55.47
Part time	56	11.27	23	9.20	33	13.36
Unemployed	31	6.24	18	7.20	13	5.26
Student	36	7.24	15	6.00	21	8.50
Other	13	2.62	8	3.20	5	2.02
Homemaker	23	4.63	8	3.20	15	6.07
Retired	33	6.64	19	7.60	14	5.67
Transgender						
Yes	25	5.03	14	5.60	11	4.45
No	468	94.16	232	92.80	236	95.55
Missing	4	0.80	4	1.60	0	0.00
Sexual orientation						
Heterosexual or straight	323	64.99	167	66.80	156	63.16
Gay or Lesbian	80	16.10	38	15.20	42	17.00
Bisexual	62	12.47	29	11.60	33	13.36
Asexual	12	2.41	7	2.80	5	2.02
Queer	19	3.82	11	4.40	8	3.24
Missing	31	6.24	17	6.80	14	5.67
Not listed	1	0.20	0	0.00	1	0.40
Psychiatric medications						
Yes	184	37.02	93	37.20	91	36.84
No	309	62.17	155	62.00	154	62.35
Missing	4	0.80	2	0.80	2	0.81
Other mental health treatment						
Yes	204	41.05	104	41.60	100	40.49
No	288	57.95	142	56.80	146	59.11
Missing	5	1.01	4	1.60	1	0.40
Smartphone operating system						
iOS (iPhone)	326	65.59	172	68.80	154	62.35
Android	171	34.41	78	31.20	93	37.65
Other	0	0.00	0	0.00	0	0.00

	M	SD	M	SD	M	SD
Mean age	44.02	13.93	43.36	13.32	44.67	14.50
Mean personal income	66221.42	57613.10	62937.00	56082.49	69479.35	59024.76
Median personal income	57000.00	–	52050.00	–	60000.00	–
Mean household income	114410.28	95139.49	110207.72	87288.91	118578.95	102341.10
Median household income	91500.00	–	90000.00	–	99500.00	–
Household size	2.56	1.36	2.62	1.39	2.51	1.32

**Table 2:** Descriptive statistics.

Baseline	SCT+HABITS		SCT	
	M	SD	M	SD
Practice automaticity	5.59	4.52	5.19	3.91
Self-compassion	65.79	11.09	65.65	12.33
Self-compassion automaticity	4.58	1.73	4.53	1.64
Stress	20.48	7.50	20.15	7.78
Symptoms of psychopathology	14.86	8.26	14.57	8.65

3-month follow-up	SCT+HABITS		SCT	
	M	SD	M	SD
Practice frequency	6.48	4.11	4.84	3.55
Practice automaticity	17.92	9.24	14.10	8.07
Self-compassion	70.43	11.91	68.68	12.65
Self-compassion automaticity	5.50	1.75	5.11	1.62
Stress	18.13	8.22	18.19	7.74
Symptoms of psychopathology	13.17	8.20	13.36	7.96

6-month follow-up	SCT+HABITS		SCT	
	M	SD	M	SD
Practice frequency	6.73	10.93	5.04	5.65
Practice automaticity	18.10	10.10	15.68	9.23
Self-compassion	71.97	12.23	70.15	12.86
Self-compassion automaticity	5.83	1.81	5.51	1.75
Stress	17.00	7.94	17.46	7.99
Symptoms of psychopathology	12.02	7.67	13.06	8.55
Acceptability	4.17	0.70	4.00	0.80

### Aim 1: condition-by-timepoint interaction contrasts for primary outcomes

Condition-by-timepoint interaction contrasts for the primary outcomes—practice frequency and practice automaticity—are summarized in Table 3. Using conventional heuristic benchmarks for standardized coefficients, significant effects for the primary outcomes were small to medium in magnitude. For practice frequency, baseline was coded as 0 because participants had not yet been introduced to the specific self-compassionate touch practice used in the study. Consistent with Hypothesis 1, participants in the SCT+HABITS group reported significantly higher practice frequency compared to the SCT group at both 3-month follow-up and 6-month follow-up, relative to this shared baseline value of zero. In line with the same hypothesis, practice automaticity was also significantly greater in the SCT+HABITS group compared to the SCT group at 3-month follow-up and 6-month follow-up.

**Table 3:** Condition-by-timepoint interaction contrasts for SCT+HABITS vs. SCT outcomes (aims 1 and 2).

	β	95% CI	*p*
**Primary outcomes**			
*** Practice frequency***			
3-month follow-up	0.27	[0.06, 0.47]	0.011
6-month follow-up	0.28	[0.07, 0.48]	0.008
*** Practice automaticity***			
3-month follow-up	0.35	[0.18, 0.52]	<0.001
6-month follow-up	0.21	[0.04, 0.37]	0.014
**Secondary outcomes**			
*** Self-compassion***			
3-month follow-up	0.14	[0.00, 0.28]	0.048
6-month follow-up	0.16	[0.02, 0.30]	0.024
*** Self-compassion automaticity***			
3-month follow-up	0.19	[0.04, 0.34]	0.012
6-month follow-up	0.17	[0.02, 0.31]	0.026
*** Stress***			
3-month follow-up	-0.06	[-0.22, 0.10]	0.471
6-month follow-up	-0.11	[-0.26, 0.05]	0.172
*** Symptoms of psychopathology***			
3-month follow-up	-0.11	[-0.25, 0.04]	0.150
6-month follow-up	-0.18	[-0.32, -0.04]	0.014

*Note: β* refers to the standardized coefficient in units of standard deviation.

### Aim 2: Condition-by-timepoint interaction contrasts for secondary outcomes

Table 3 presents condition-by-timepoint interaction contrasts for the secondary outcomes: self-compassion, self-compassion automaticity, stress, and symptoms of psychopathology. Using conventional heuristic benchmarks for standardized coefficients, significant effects for the secondary outcomes were small in magnitude. In keeping with Hypothesis 2, at 3-month follow-up and 6-month follow-up, the SCT+HABITS group showed significantly greater increases in both self-compassion and self-compassion automaticity compared to the SCT group. No significant differences were found between groups for perceived stress at either 3-month follow-up or 6-month follow-up. Symptoms of psychopathology scores did not significantly differ between groups at 3-month follow-up. In accordance with Hypothesis 2, at 6-month follow-up, the SCT+HABITS group showed a significantly greater reduction in symptoms of psychopathology compared to the SCT group.

### Aim 3: Baseline to 3-month follow-up changes in practice automaticity as mediator of group differences in outcomes at 6-month follow-up

Table 4 presents the results of the mediation analysis, which examined whether improvements in practice automaticity from baseline to 3-month follow-up mediated the effect of SCT+HABITS relative to SCT alone on self-compassion, self-compassion automaticity, perceived stress, and symptoms of psychopathology at 6-month follow-up.

**Table 4:** Baseline to 3-month follow-up change in practice automaticity as mediator of group differences in outcomes at 6-month follow-up (aim 3).

Baseline to 6-month follow-up change in outcome	*R*²	Indirect effect (95% CI)	Direct effect (95% CI)	Total effect (95% CI)
Self-compassion	0.45	0.07 (0.04, 0.11)	0.02 (-0.06, 0.08)	0.09 (0.01, 0.16)
Self-compassion automaticity	0.39	0.08 (0.04, 0.12)	0.02 (-0.06, 0.09)	0.09 (0.01, 0.18)
Stress	0.35	-0.03 (-0.06, -0.01)	-0.02 (-0.10, 0.06)	-0.05 (-0.13, 0.02)
Symptoms of psychopathology	0.39	-0.03 (-0.06, -0.01)	-0.05 (-0.12, 0.03)	-0.08 (-0.16, -0.01)

Note: All effects are standardized coefficients in units of standard deviation.

Consistent with Hypothesis 3, baseline to 3-month follow-up improvements in practice automaticity significantly mediated the effect of SCT+HABITS relative to SCT alone on self-compassion, self-compassion automaticity, and symptoms of psychopathology at 6-month follow-up. Practice automaticity significantly mediated reductions in perceived stress, though the total effect remained non-significant.

### Exploratory Aim 1: Acceptability

Participants rated the acceptability of their assigned intervention, SCT+HABITS or SCT, at 6-month follow-up. The mean acceptability score for both SCT+HABITS and SCT exceeded the pre-registered threshold for acceptability, with a mean score of > 3, reflecting favorable feedback (see Table 2)^36^.

### Exploratory Aim 2: Examination of differences in primary and secondary outcomes among non-Hispanic white and racialized participants

To investigate whether the effects of the SCT+HABITS intervention relative to SCT alone were equally beneficial for non-Hispanic white participants and racialized participants, this study examined whether the results of Aims 1 and 2 were moderated by racialized identity (yes/no). Table 5 presents the results of these analyses. No significant three-way interactions were found for practice frequency, practice automaticity, self-compassion, self-compassion automaticity, stress, or symptoms of psychopathology at either follow-up time point, indicating that intervention outcomes did not significantly differ based on whether or not the participant identified as non-Hispanic white.

**Table 5:** Three-way interaction of condition, timepoint, and racialized identity (exploratory aim 2).

	β	95% CI	*p*
**Primary outcomes**			
*** Practice frequency***			
3-month follow-up	-0.30	[-0.77, 0.16]	0.198
6-month follow-up	-0.40	[-0.86, 0.05]	0.084
*** Practice automaticity***			
3-month follow-up	-0.13	[-0.51, 0.24]	0.487
6-month follow-up	0.13	[-0.24, 0.50]	0.483
**Secondary outcomes**			
*** Self-compassion***			
3-month follow-up	-0.11	[-0.42, 0.20]	0.488
6-month follow-up	0.06	[-0.25, 0.37]	0.713
*** Self-compassion automaticity***			
3-month follow-up	-0.04	[-0.37, 0.29]	0.814
6-month follow-up	0.18	[-0.15, 0.51]	0.278
*** Stress***			
3-month follow-up	0.15	[-0.20, 0.51]	0.399
6-month follow-up	0.12	[-0.23, 0.47]	0.501
*** Symptoms of psychopathology***			
3-month follow-up	-0.07	[-0.40, 0.25]	0.653
6-month follow-up	0.12	[-0.21, 0.44]	0.478

*Note: β* refers to the standardized coefficient in units of standard deviation. The coefficients reflect how outcomes for non-Hispanic white participants differ from those of racialized participants across conditions and timepoints: positive coefficients indicate higher scores for non-Hispanic white participants relative to racialized participants, while negative coefficients indicate lower scores for non-Hispanic white participants relative to racialized participants.

## Discussion

This study evaluated a 15-minute, self-guided, online, single-session habit formation intervention aimed at promoting the micropractice of self-compassionate touch in a national adult sample recruited through social media. The intervention builds on an ongoing habit formation study conducted in a different population^30,31^ and aligns with recommendations to integrate habit formation science into evidence-based interventions^14^. Pre-registered analyses revealed several key results.

With respect to Aim 1, SCT+HABITS significantly increased both practice frequency and practice automaticity at 3-month follow-up and 6-month follow-up compared to SCT. Although prior research suggests that self-guided online interventions are less effective at establishing a persistent well-being practice compared to those involving human contact^9^, the present findings demonstrate that pairing self-guided online interventions with habit formation tools may mitigate this limitation. Notably, this study adds to the growing evidence that such interventions do not need to be time-intensive; even a single 15-minute session delivered in a self-guided online format can yield significant and enduring behavior change^36,37^.

In terms of Aim 2, compared to SCT, SCT+HABITS showed significantly greater increases in self-compassion and self-compassion automaticity at 3- and 6-month follow-ups. Together, these findings indicate that SCT+HABITS may offer a promising approach for supporting more consistent and less effortful engagement in self-compassion.

SCT+HABITS also led to significantly greater reductions in symptoms of psychopathology at 6 months, but not at 3 months, relative to SCT. This pattern aligns with evidence that frequent engagement in brief touch-based practices predicts improved emotional well-being over time^10,13,38^. The results also support growing evidence that integrating habit formation science can bolster the longer-term impact of well-being interventions^14,30,31,39^.

SCT+HABITS did not lead to greater reductions in stress compared to SCT. Although frequent self-compassionate touch predicted lower stress in prior work, differences in study context may help explain this discrepancy^10^. The earlier SCT study occurred during COVID-19-related social restrictions^10^, a period marked by heightened touch avoidance^40^—conditions under which touch-related practices may exert stronger effects on stress.^41^ Additionally, whereas the prior study evaluated SCT compared to a finger-tapping active control^10^, the current study compared SCT to itself plus habit formation tools. These are fundamentally different comparisons, and the increased practice frequency facilitated by the habit tools may not have been sufficient to produce a significantly greater reduction in stress. Future research with larger samples and more frequent assessments may help unravel these complexities and pinpoint the conditions where self-compassionate touch is most effective for stress relief.

Moving to Aim 3, the mediation analyses showed that improvements in practice automaticity from baseline to 3-month follow-up accounted for approximately 40–83% of the total effects of SCT+HABITS, relative to SCT, on improvements in self-compassion, self-compassion automaticity, and symptoms of psychopathology from baseline to 6-month follow-up. For stress, practice automaticity showed a significant indirect effect despite a non-significant total effect. This pattern suggests that SCT+HABITS may not have directly reduced stress but may have worked indirectly by strengthening the habit of self-compassionate touch, which in turn predicted stress reduction. Elevated stress can hinder habit formation, which may have diminished the effectiveness of habit tools for those with the greatest room to benefit from stress reduction^42,43^. However, it’s also possible that the study had sufficient power to detect the indirect pathway but not a small direct effect^44^. Nevertheless, these results support the preregistered hypothesis that integrating habit-formation tools into self-compassionate touch enhances its impact by making practice initiation more automatic over time, and they add to evidence that the benefits of well-being practices often rely on their establishment as habits^10,11,45^.

For Exploratory Aim 1, both SCT+HABITS and SCT exceeded the preregistered acceptability threshold, indicating that both interventions were well-received and user-friendly^36^. These findings highlight the potential of SCT+HABITS and SCT as accessible approaches to supporting emotional well-being^6,36^.

For Exploratory Aim 2, outcomes did not significantly differ between SCT+HABITS and SCT based on racialized identity, suggesting comparable benefits for non-Hispanic white and racialized participants. However, given that approximately 62% of the English-speaking U.S. population identifies as non-Hispanic White—and the present sample was 71% non-Hispanic White—replication with a larger, more racially diverse sample is needed to more definitively assess effectiveness across demographic groups^46–48^.

As with any study, there are limitations and constraints on generality. First, by conventional heuristic benchmarks, many of the effect sizes for condition-by-timepoint interaction contrasts in the present study were small in magnitude, particularly for secondary outcomes. That said, several features of the study suggest these effects may be practically meaningful. The comparator is important: SCT+HABITS was compared with SCT itself, a higher bar than comparisons with inactive or waitlist controls, which are known to yield larger effects than active or head-to-head comparisons that tend to produce small effects^49,50^. The 3- and 6-month follow-up periods and self-guided format also warrant consideration. A meta-analysis found that single-session intervention effects were larger at 0- to 2-week follow-up (*g* = 0.46) than at follow-ups of 3 months or longer (*g* = 0.07), and smaller for self-guided (*g* = 0.21) than human-delivered (*g* = 0.33) single-session interventions^37^. Therefore, the present effects should be interpreted in light of the study’s self-guided format, longer follow-up period, and active comparator.

The behavioral impact also appears to be meaningful: although standardized practice-frequency effects were small-to-moderate, they corresponded to nearly two additional practice sessions per week in SCT+HABITS relative to SCT alone—roughly 50 additional practice episodes over six months, or approximately one-third more practice than SCT alone. Thus, even modest standardized differences reflected a substantial increase in cumulative exposure to the micropractice over time. More broadly, methodological discussions caution against interpreting effect sizes solely through generic benchmarks, noting that effects conventionally labeled “small” may be meaningful when interventions are low-cost, low burden, scalable, and applied to consequential public health outcomes^51–54^. Therefore, although the observed effects should not be overstated, their persistence over six months, low burden, scalability, and incremental benefit over an active self-compassion intervention suggest practical and public-health relevance. Future research should evaluate whether these effects replicate and whether changes of this magnitude correspond to clinically meaningful improvement for individuals.

A second limitation is that, although participants could not skip past the instructional videos, the absence of attention or comprehension checks related to the instructional videos prevented verification of participants’ engagement with the video content. Third, while the assessments of self-reported practice frequency lack the precision of ecological momentary assessment (EMA), they reliably align with actual behavior across settings^55–58^. Self-reported practice frequency was used because EMA has been shown to alter participant behaviors, and preserving naturalistic behavior was essential to the study’s aims^59–61^. Nevertheless, replications using EMA could help validate findings and provide a more granular window into the trajectories of SCT+HABITS’ impact on behavior.

Fourth, although the present findings support the use of a single-session, self-guided habit-formation intervention to strengthen self-compassionate touch micropractice specifically, its generalizability to other interventions remains an important question. We hypothesize that the habit-formation intervention tested here may be especially well-suited to practices that are brief, simple, and easily paired with daily cues. Future research is needed to determine whether habit tools complement or strengthen other micropractices, well-being practices, psychological therapies, and behavioral interventions, and, if so, which tools or combinations of tools are most effective under which conditions. Finally, the findings may not generalize to populations without access to smartphones, a limitation more prevalent among older adults, low-income groups, and rural residents^62^. Although the median personal income and age of the study sample were comparable to the United States adult population^63,64^, future research should explore whether low-tech alternatives, like notes on a bathroom mirror, could offer comparable benefits to smartphone reminders and enhance accessibility for underserved populations.

Findings from this preregistered study demonstrate that integrating evidence-based habit-formation tools with self-compassionate touch enhances both habit formation and psychological outcomes. Notably, these improvements resulted from just one 15-minute session, with benefits persisting up to six months. Establishing self-compassionate touch as a habit appeared to play a key role in these effects. Based on a national sample of adults, these results underscore the effectiveness of self-compassionate touch in enhancing self-compassion and emotional well-being. They also contribute to a growing body of research highlighting the central role habits play in the benefits of well-being practices^10,11,45^ and psychological interventions^14,39,65^. While further research is needed to confirm practice automaticity as a mechanism of change in psychological interventions^66^, the present findings strengthen support for this possibility. Its scalability, low cost, and potential adaptability to other practices position SCT+HABITS as a promising model for advancing well-being interventions. While not a substitute for professional mental health care, SCT+HABITS offers a practical, user-friendly approach to enhancing self-compassion and reducing symptoms of psychopathology, providing an accessible pathway to improved well-being for diverse populations.

## Method

### Design

We randomly assigned participants to SCT or SCT+HABITS. Both groups completed online assessments at baseline, 3-month follow-up, and 6-month follow-up. Participants were instructed to practice self-compassionate touch at least once per day through 6-month follow-up. The study is reported in accordance with the CONSORT statement for nonpharmacological trials^67^. All analyses were preregistered (osf.io/5uds4), and all data, materials, and analysis code are publicly available on the Open Science Framework (osf.io/3hcqv). The protocol was registered at ClinicalTrials.gov (clinicaltrials.gov/ct2/show/NCT05866718). This study was approved by the Ethics Committee of the University of California, Berkeley, and all methods were performed in accordance with relevant guidelines and regulations. Informed consent was obtained from all participants.

### Participants

The pre-registered plan was to recruit a nationwide sample of as many participants as the budget allowed. The target was to recruit 440 adults from the general population, ages 18+, throughout the United States via social media postings and advertisements. Ultimately, 497 adults were successfully recruited across the United States between June 2023 and February 2024.

To indicate interest in participating in the study, participants were provided a link in the posting that directed them to an eligibility screener questionnaire on Qualtrics. Following their completion of this survey, they received an email informing them whether they were eligible to participate. If they were eligible, they received a link to the baseline survey in this email. After completing the baseline survey, participants received links to complete their 3-month follow-up and 6-month follow-up surveys three and six months later, respectively. All procedures were delivered online via Qualtrics at a location of the participant’s choosing. Inclusion criteria were: (a) 18 + years of age; (b) English language proficiency; (c) able/willing to give informed consent; and (d) resides in the United States of America. Exclusion criteria were: (a) no email address or access to email; (b) not able/willing to participate in and/or complete the baseline assessments; and (c) does not personally own a smartphone device. Power analysis with the *pwr* package in R suggests that 497 participants are sufficient to identify small effects (Cohen’s *d* = 0.25) via a two-sample t-test at 80% power and *p* < .05 significance level. Accounting for actual attrition, 446 participants remain sufficient to identify small effects (Cohen’s *d* = 0.27) via a two-sample t-test at 80% power and *p* < .05 significance level. We had originally planned to recruit at least 440 participants because after 20% attrition, 375 participants would be sufficient to identify small effects (Cohen’s *d* = 0.29) via a two-sample t-test at 80% power and *p* < .05 significance level. Given that prior research on self-compassionate touch showed small to moderate conditional effects of practice frequency on outcomes^10^, it was expected that at least 440 participants, including attrition, would be adequate.

### Data quality and bot-detection procedures

Several procedures were used to protect data quality across the eligibility screener and online study surveys. The eligibility screener and surveys included CAPTCHAs and Qualtrics bot-detection features, including reCAPTCHA v3 bot detection, fraud detection, duplicate detection, prevention of multiple submissions, security scan monitoring, and search-engine non-indexing^68,69^. Eligible participants were also provided unique, one-time-use survey links to further reduce the likelihood of duplicate or bot responses^70^. Responses were screened for implausible or internally inconsistent information (e.g., self-reported birthdate not matching self-reported age)^71^. In addition, participants completed empirically supported bot-screening questions adapted from prior work on psychological methods for bot detection^72^. These questions were designed to identify bots while minimizing burden and remaining straightforward for valid human respondents^72^, and their item order and correct responses were randomized to prevent the use of a fixed answer key. Finally, recruitment advertisements did not disclose financial compensation^73^, and survey links included in advertisements were obfuscated using a URL shortener (bit.ly) to reduce the likelihood that automated systems would identify the study as a compensated online survey.

### Randomization

To ensure unbiased allocation, participants were randomly assigned to one of the study’s two conditions (SCT or SCT+HABITS) in a 1:1 parallel group design using the computer-generated randomizer in Qualtrics. There was no direct interaction between study personnel and participants during this process. To safeguard against cross-condition contamination, participants were asked to provide a written commitment not to share any study materials with others. Furthermore, participants were kept blind to the existence of two conditions; all were informed that they were receiving the intervention without any indication of alternative conditions.

### Procedure

At the baseline assessment, participants first completed the outcome and other measures and then were randomized to receive the SCT or SCT+HABITS intervention. At all assessments, participants completed the measures in the same order.

### Intervention

All participants received the self-compassion intervention after completing their baseline assessment, which contained the same instructions used by prior research^10^ (See Supplementary Note 1). The self-guided, online intervention was composed of three parts. First, participants were taught the micropractice via video recording. In the video, participants were guided through a practice of self-compassionate touch. The instructions can be found in Supplementary Table 1. Second, participants were taught to choose a cue to precede their daily use of the micropractice. Participants were provided examples to guide them in selecting their optimal cue (“When I finish brushing my teeth, I will practice the engaging touch exercise.”). Participants documented the cue they chose and were emailed a record of their selected cue, along with the recording and transcript of the self-compassion micropractice that they could refer back to for guidance. Third, participants were asked to practice at least once per day, following their chosen cue, for six months until they completed their final assessment (6-month follow-up). One study estimated that habit formation of new behaviors takes between 18 and 254 days to fully take hold^74^. Thus, six months may allow a more accurate window for evaluating differences in habit formation between SCT+HABITS and SCT.

### Habit formation tools

SCT+HABITS participants received the abovementioned procedures and then were immediately shown a self-guided, online video presentation explaining the concept of habit formation, including (a) evidence-based advice for selecting the optimal cue to prompt the start of the micropractice; (b) an emphasis that repetition in a consistent context leads to habit formation through associative learning^74^; (c) identifying intrinsic rewards to build positive associations with the new self-compassionate touch habit^75 ^(See Supplementary Note 2 for script of video). After watching the video, participants filled out in Qualtrics a detailed plan about (i) the times they will practice each day, (ii) setting daily phone alerts at that time to remind them to practice, (iii) where they will practice each day, (iv) identifying the cue to prompt the start of the micropractice, (v) overcoming barriers to practicing, and (vi) identifying the rewards of performing self-compassionate touch.

According to Schleider and Weisz^37^, “interventions are defined as ‘single-session’ if they involved just one visit or encounter with a clinic, school, or program (p. 108).” Therefore, instructing the participant to set a daily reminder as part of their single encounter falls under this definition of “single-session.”

Participants were also told that when they really could not squeeze in practicing once per day, they were welcome to give themselves up to two emergency “skip days” per week, based on research demonstrating that “skip days” better promote daily practice habits^76^. Lastly, participants were shown a copy of their detailed plan and instructed to close their eyes and visualize each step of performing their plan^77^. Participants were emailed a copy of their plan and the video presentation for reference. These strategies follow an existing ongoing study conducted with a different population as well as recommendations for infusing the science of habit formation into evidence-based interventions^14,30,31^. Together, they are designed to optimize habit formation and address the specific challenges of single-session, self-guided online interventions for contemplative practice.

### Measures

Primary and secondary outcomes and other measures are registered at ClinicalTrials.gov (clinicaltrials.gov/ct2/show/NCT05866718). The primary outcomes were selected for relevance to evaluating the efficacy of SCT+HABITS relative to SCT. The secondary outcomes are additional measures of interest for evaluating the effects of SCT+HABITS relative to SCT based on evidence that more frequent practice of self-compassionate touch was associated with greater increases in self-compassion and greater reductions in stress and symptoms of psychopathology, as well as an interest in evaluating self-compassion automaticity over a longer timeframe than in the previous study^10^. The other measures are to evaluate the acceptability, barriers, and facilitators of the self-compassionate touch practice and to test potential moderator effects of the confirmatory aims. Participants’ demographics were also collected. The schedule of measures is described in Supplementary Table 2.

#### Primary outcomes

***Practice frequency.*** Practice frequency was assessed by asking participants to report on the number of times per week that they performed the micropractice over the course of the period since their last assessment.

***Practice automaticity.*** In all assessments, this study utilized the Self-Report Behavioral Automaticity Index (SRBAI) to gauge the degree to which one initiates self-compassionate touch without deliberation^32^. The SRBAI is a validated measure that is generally used to assess habit formation by indexing the extent to which a behavior (e.g., initiating the practice of SCT) is elicited with minimal deliberation. Although the SRBAI has been criticized for relying on subjective evaluations of automaticity^78^, recent work supports its validity through significant associations with objective laboratory indices of habit, such as Go/No-go tasks where participants habitually responded to previously rewarded cues even when rewards were devalued^79^. It contains four items rated on a 1 (“Strongly Disagree”) to 9 (“Strongly Agree”) scale, where higher scores indicate greater habit formation (e.g., a “5” indicates moderate habit formation): [“Behavior X is something…”] (1) “I do automatically,” (2) “I do without having to consciously remember,” (3) “I do without thinking,” and (4) “I start doing before I realize I’m doing it”) where, following standard procedure, “Behavior X” refers to the construct of interest^32^. This measure has demonstrated excellent reliability in evaluating the practice automaticity of micropractices (Cronbach’s α = 0.95)^10^. During the baseline assessment, participants were instructed, as in previous studies^10^, to select the lowest score (“1 (Strongly Disagree)”) if they had never tried SCT before enrollment in this study.

#### Secondary outcomes

***Self-compassion.*** This study assessed self-compassion using the Sussex-Oxford Compassion for the Self Scale (SOCS-S)^15^. Gu et al.^15^ developed the SOCS-S based upon a robust empirically and theoretically supported conceptualization of (self-) compassion. The SOCS-S is considered a significant advancement in the operationalization of self-compassion because it covers all elements considered pertinent to the construct, aligning with a range of theories and definitions^16,80,81^. SOCS-S was also selected for its suitability in studies examining symptoms of psychopathology, as it has been proposed to be the most appropriate measure for assessing self-compassion in this context^80,81^. The SOCS-S comprises five subscales, each containing four items, corresponding to the five components of self-compassion outlined in the introduction (20 items, e.g., “When I’m upset, I do my best to take care of myself,” 5-point response scale, Cronbach’s α = 0.95)^15^.

***Self-compassion automaticity.*** To assess current self-compassion automaticity, which indicates the reflexive spontaneity of compassionate responses towards oneself in daily life, this study employed the Self-Report Behavioral Automaticity Index^32^. As described above, this index consists of four items structured around statements like “Behavior X is something.” where, following standard procedure, “Behavior X” denotes the construct of interest^32^. For self-compassion automaticity, this measure included five “Behavior Xs,” each representing one of the five elements of self-compassion, forming a 20-item SRBAI. For each of the five “Behavior Xs,” the item with the highest factor loading from the SOCS-S validation sample was selected^15^. To ensure the sentences were grammatical, minor grammar adjustments were made to these five SOCS-S items without altering their meaning. The five “Behavior X” question stems were: 1) “Noticing when I’m feeling distressed is something…”, 2) “Remembering that everyone experiences suffering at some point in their lives is something…”, 3) “When I’m going through a difficult time, feeling kindly towards myself is something…,” 4) “Connecting with my own suffering without judging myself is something…”, and 5) “When I’m going through a difficult time, trying to look after myself is something…”. The five items employed as “Behavior X” and their corresponding component of self-compassion are shown in Supplementary Table 3. The mean of the five items was used as the score for total self-compassion automaticity.

***Perceived stress.*** Perceived stress in the past week was assessed using the Perceived-Stress Scale (10 items, e.g., “In the last week, how often have you felt nervous and ‘stressed’?”, 5-point response scale, Cronbach’s α = 0.84)^82^.

***Symptoms of psychopathology.*** Symptoms of psychopathology over the past two weeks were indexed using the DSM-5 Cross-Cutting Measure (22 items, e.g., “Little interest or pleasure in doing things?”, 5-point scale)^83^. As suggested and psychometrically validated by Mahoney et al.^84^, the thoughts of self-harm item (Q11) was omitted from the assessment. This decision was made because participants completed the measure online, and real-time response monitoring is not feasible. Therefore, timely follow-up for individuals indicating such thoughts would not be possible.

#### Other measures

***Acceptability and feasibility.*** Acceptability was measured using the Program Feedback Scale (PFS)^36^. This scale was developed specifically for evaluating online single-session interventions and was based on validated acceptability measures of digital interventions. At the six-month follow-up, participants rated agreement with seven statements indicating perceived acceptability and feasibility of their single-session intervention (e.g., “I enjoyed the program”) on a 5-point Likert scale (1=“really disagree”; 5=“totally agree”). PFS item scores may be evaluated individually or via a mean score across items.

***Race and ethnicity.*** Race and ethnicity were assessed via self-report using a checkbox format, allowing participants to select one or more categories. Participants could indicate American Indian or Alaska Native, Asian, Black/African American, Hispanic/Latinx, Native Hawaiian or Other Pacific Islander, White, Prefer not to say, or Not listed above (with a write-in option). Participants who selected only White (without selecting any other racial category or Hispanic/Latinx) were categorized as non-Hispanic white. Participants identifying as Hispanic/Latinx, American Indian or Alaska Native, Asian, Black/African American, Native Hawaiian or Other Pacific Islander, multiracial, or with a self-described racial identity were categorized as racialized for Exploratory Aim 2.

## Data analysis

We conducted analyses in R Version 4.1.2. Hypotheses 1–2 were evaluated using multi-level modeling, with outcome variables modeled continuously. Models used restricted maximum likelihood and assumption of missing at random to evaluate the outcomes. The fixed part included a condition code (0 = SCT, 1 = SCT+HABITS), a pseudo-coded timepoint (0 = baseline, 1 = 3-month follow-up, 2 = 6-month follow-up), and a timepoint-by-condition interaction term. The random part included a subject-specific random intercept. We used categorical timepoint rather than continuous time for both substantive and analytic reasons. Substantively, the preregistered hypotheses for Aims 1 and 2 concerned whether SCT+HABITS showed greater baseline-to-month-3 and baseline-to-month-6 changes than SCT, rather than whether there was a single linear effect of time across conditions. Analytically, with three assessment occasions, modeling time continuously would impose a linear change assumption, whereas the rate of change may differ from baseline to 3-month follow-up versus from 3-month to 6-month follow-up. Modeling timepoint categorically relaxes this assumption and allows the baseline-to-month-3 and baseline-to-month-6 *condition-by-timepoint* contrasts to be estimated separately^85^. For practice frequency, baseline values were coded as 0 because participants had not yet been introduced to the specific self-compassionate touch practice used in the study. This coding reflected the study design and preregistered analytic plan. Because the target behavior was newly introduced during the intervention, all participants shared the same baseline value for practice frequency, and follow-up values represented subsequent engagement with the study-specific practice. Accordingly, the practice-frequency contrasts in Table 3 represent between-condition differences in practice frequency at 3-month and 6-month follow-up relative to this shared baseline value of zero. Analyses followed an intention-to-treat approach, with participants analyzed according to randomized assignment rather than intervention completion status. All randomized participants remained eligible for follow-up assessments and were analyzed according to randomized assignment, with all available data included in the models.

We evaluated Hypothesis 3 using structural equation modeling^86^. Bootstrapping with 5,000 replications tested the statistical significance of the indirect effect from group to outcome via the proposed mediator (practice automaticity). If SCT+HABITS does not show superior baseline-to-month-6 changes on a given outcome (i.e., self-compassion, self-compassion automaticity, stress, or symptoms of psychopathology), the abovementioned mediation analysis for that outcome variable would not be conducted. If baseline-to-month-3 increases in practice automaticity do not mediate baseline-to-month-6 increases in self-compassion and self-compassion automaticity and reductions in stress and symptoms of psychopathology, practice frequency at month 3 would be examined as a mediator of baseline-to-month-6 increases in self-compassion and self-compassion automaticity, and reductions in stress, and symptoms of psychopathology. Practice frequency was hypothesized to mediate these associations.

For the exploratory aim of examining the conditions’ acceptability, means and standard deviations for each PFS item and for the overall PFS score were reported. As described in Schleider et al.^36^, mean scores of > 3 on any PFS item reflect item endorsement (i.e., adequate feasibility/positive feedback). A mean overall PFS score of > 3 across all items indicates the intervention’s overall perceived acceptability.

To assess whether the effects observed in Aims 1 and 2 were not limited to non-Hispanic white participants, three-way interactions among condition, timepoint, and non-Hispanic white identity (non-Hispanic white = 1, racialized participants = 0) were examined for predicting primary and secondary outcomes. For each outcome, models included non-Hispanic white Identity as a moderator, with participant-specific random intercepts. This analysis aimed to examine whether the SCT+HABITS intervention provided equitable benefits to racialized participants, ensuring that the intervention’s positive effects were inclusive and beneficial for racialized participants. Moderated mediation analyses for Aim 3 were neither conducted nor pre-registered. Without differential intervention effects, mediation effects would not be expected to differ meaningfully.

Given that all tests conducted were for separate, a priori hypotheses, there was no correction for multiple comparisons. Some statisticians suggest that in the case of a priori specification, correcting for multiple comparisons may not be necessary and could be counterproductive^87,88^. If it were specified a priori to correct for multiple comparisons, a larger sample would have been needed to maintain sufficient power to detect small effect size differences. However, unnecessarily increasing the sample size for an intervention (i.e., SCT+HABITS) that has yet to be empirically tested is generally discouraged^89^. Given that the hypotheses, analyses, and inference criteria were all specified a priori, this study aimed to keep in good faith with its pre-registration and not correct for multiple comparisons. This approach was intended to avoid unnecessarily increasing the risk of Type II error or introducing publication bias^89^. Thus, to account for multiple comparisons, the analyses were pre-registered based on existing empirical literature and theory, and this study is already being more conservative by using two-sided tests even though the analyses are pre-registered. Additionally, this study emphasizes interpreting the degree of certainty of the estimates by reporting standardized effect sizes and 95% confidence intervals in all analyses^89^. Nevertheless, another method to address controlling for Type I error is replication and multiple experiments to confirm the results, so future research should attempt to replicate the findings from this study.

### Calculation of effect sizes

In the multilevel models, standardized coefficients were calculated by standardizing all continuous variables so the regression coefficients can be interpreted as effect sizes in standard deviation units^90^.

## Supplementary Information

Below is the link to the electronic supplementary material.

Supplementary Material 1

## Data Availability

The datasets generated during and/or analyzed during the current study are available in the Open Science Framework (OSF) repository, https://osf.io/3hcqv.
