# Supplementary material for: Habit-formation strategies enhance long-term outcomes of a self-compassionate touch micropractice: a randomized controlled trial
Source: Sci Rep. 2026 Sep 15;16:25100. doi: 10.1038/s41598-026-63457-4 (PMC13578738; doi:10.1038/s41598-026-63457-4)
Supplement: Supplementary file 1 [file 41598_2026_63457_MOESM1_ESM.pdf]

Supplementary Information

Habit-Formation Strategies Enhance Long-Term Outcomes of a Self-Compassionate Touch Micropractice: A Randomized Controlled Trial

Eli S. Susman, MA[1], Serena Chen, PhD[1], Dacher Keltner, PhD[1], Allison G. Harvey, PhD[1]

[1] University of California, Berkeley, Department of Psychology, 2121 Berkeley Way, Berkeley, CA 94720

ClinicalTrials.gov Identifier: NCT05866718, registered May 23, 2023.

Corresponding Author:

Eli S. Susman, University of California, Berkeley, Department of Psychology, 2121 Berkeley Way, Berkeley, CA 94720.

Email: esusman@berkeley.edu

Email addresses for Co-authors: serchen@berkeley.edu, keltner@berkeley.edu, aharvey@berkeley.edu

Funding: The National Science Foundation Graduate Research Fellowship (grant number DGE2146752 to E.S.S.), Mind and Life Institute (E.S.S.), Greater Good Science Center (E.S.S.), and the National Institute of Child Health and Human Development (grant number R01HD071065 to A.G.H.) supported this work. The funding agencies have no role in the conduct or reporting of this study.

Supplementary Table 1. Instructions for SCT and SCT+HABITS

**Self-Compassion Intervention**
You're invited to allow your eyes to close… Bringing to mind a recent mistake, or a failure or something about yourself that has been bothering you lately, or has made you feel unworthy, unloved, or not enough... and notice what arises in your body as you bring this to mind... sending kindness and warmth to yourself by bringing one hand to your belly and the other to your chest with the energy of giving yourself a hug… allow yourself to embrace what arises in your body… and you're invited to ask yourself 'how can I be a friend to myself in this moment?'… and when you're ready you may open your eyes.

Supplementary Table 2. Schedule of Measures

	Baseline	Month 3	Month 6
Demographics	•		
Practice Frequency		•	•
Practice Automaticity	•	•	•
Self-Compassion	•	•	•
Self-Compassion Automaticity	•	•	•
Perceived Stress	•	•	•
Symptoms of Psychopathology	•	•	•
Barriers and Facilitators			•
Acceptability			•
Reminders		•	•

Supplementary Table 3. SRBAI for Self-Compassion Automaticity

1) Noticing when I'm feeling distressed is something…<br>1. I do automatically<br>2. I do without having to consciously remember<br>3. I do without thinking<br>4. I start doing before I realize I'm doing it
2) Understanding that everyone experiences suffering at some point in their lives is something…<br>1. I do automatically<br>2. I do without having to consciously remember<br>3. I do without thinking<br>4. I start doing before I realize I'm doing it
3) When I'm going through a difficult time, feeling kindly towards myself is something…<br>1. I do automatically<br>2. I do without having to consciously remember<br>3. I do without thinking<br>4. I start doing before I realize I'm doing it
4) Connecting with my own suffering without judging myself is something…<br>1. I do automatically<br>2. I do without having to consciously remember<br>3. I do without thinking<br>4. I start doing before I realize I'm doing it
5) When I'm going through a difficult time, trying to look after myself is something…<br>1. I do automatically<br>2. I do without having to consciously remember<br>3. I do without thinking<br>4. I start doing before I realize I'm doing it

Supplementary Note 1. Original Self-Compassionate Touch Intervention in Susman et al.[1]

The intervention included three parts. First, participants were guided through a practice of the self-compassionate touch via video recording (see Supplementary Table 1 for transcript and osf.io/wq8mf/for video). This video was developed based on Dreisoerner et al.[2] and Neff[3]. In Dreisoerner et al.[2], participants were shown a variety of positions for the self-comp assionate touch, the most chosen being right-hand-over-chest, left-hand-over-belly. In the present study, the video depicted left-hand-over-chest, right-hand-over-belly, but participants were encouraged to choose any method of touch that supported them in feeling kindness and warmth towards themselves. Second, participants chose a cue to precede their daily use of SCT. Participants received examples ("When I finish brushing my teeth, this will cue my use of the exercise.") to guide them in selecting their cue. Participants noted their chosen cue in Qualtrics and were emailed a record of their cue, along with the recording and transcript of SCT, that they could reference as desired. Third, participants were encouraged to practice self-compassionate touch at least once per day following their chosen cue for the next month.

Supplementary Note 2. Script of Video Shown to SCT+HABITS

Hi, my name is Eli and I'm a researcher with the Golden Bear Sleep Mood Research Clinic at UC Berkeley. First, I want to thank you for participating in the self-compassion study. Your participation is playing such a crucial role in furthering the understanding of how to foster self-compassion. As a part of this study, your instructions will be to practice self-compassionate touch, just like what you tried in the video, at least once per day for the next six months. We know firsthand how difficult it can be to develop the habit of practicing something daily, which is why I'm here to share some exciting tips and tricks for developing a daily habit of practicing self-compassionate touch, for fostering self-compassion. And if you find this information to be helpful, you're more than welcome to apply it to other habits you wish to build in your life also.

So, why are habits so important? Well, unlike goal-directed behaviors, which are consciously controlled, habits are behaviors that we do automatically and are thought to develop through repeated practice in the same context until the context can automatically cue the behavior on its own. And what's so important here is that when our habits and our goals conflict, most of the time, our habits win. So, let's say you made a mistake, or did something that made you feel unworthy, unloved, or "not enough"—if you have a strong habit of practicing self-compassionate touch, then you'll be more likely to practice it and bring self-compassion into your life, even in these tough moments when you don't necessarily feel like doing so. So today, we'll go through some tips and tricks to help you build stronger habits.

The three important components to forming habits are practicing self-compassionate touch in response to the same cues, fully experiencing and savoring the rewards of that self-compassionate touch, and practice, practice, practice. So, to develop a habit, we have to set up cues and rewards and then practice.

Practice is one of the most important ingredients of habit formation. In this study, we'll try daily practice over the next six months. And although your instructions will be to practice once per day, when you really can't squeeze it in, this is an opportunity to practice compassion towards yourself. So, feel free to give yourself up to two emergency skip days per week in case you need it. And finally, habits don't happen overnight. If you notice it to be challenging to practice regularly in the first few weeks, don't give up. It may take several weeks before it starts to become part of your routine. And the more you do it, the easier it will get.

The next ingredient of habit formation is the cue. Practicing self-compassionate touch after the same cue every time you do it is essential for habit formation. As people repeat self-compassionate touch after the same cue, their intentions and goals to perform the behavior gradually become less influential, whereas their habit impulse to practice self-compassionate touch becomes more influential. For example, let's say you were to practice self-compassionate touch every time after you finish brushing your teeth. Over time, the more you do this, the more brushing your teeth will automatically start cueing your practice of self-compassionate touch without your even thinking about it. However, if you realize you forgot to practice it with your cue, no problem! It's fine to practice whenever you remember, because even though it's not paired with your cue, more practice never hurts!

The self-compassion practice should also occur after the cue. For example, practicing self-compassionate touch after putting on your pajamas in your bedroom at night would work because putting on your pajamas would then cue the practice. However, practicing self-compassionate touch with a cue of before getting into bed wouldn't work because there's not anything to prompt the self-compassion practice. If this is the time you would like to practice self-compassionate touch, think about what you're actually doing before bed and make that the

cue to practice self-compassionate touch. For example, in addition to putting on your pajamas, other examples of cues at bedtime could be turning off the light at bedtime, putting on your face lotion, or putting your phone in its charger.

The ideal cues for practicing self-compassionate touch are often things that you encounter every day. It may be helpful to choose something that you already do habitually as part of your routine, like brushing your teeth, washing your hands, getting into your car, or getting out of bed. If you're not stressed very often, don't choose stress as your cue. If you often forget to brush your teeth, don't choose brushing your teeth as your cue. Your cue to practice self-compassionate touch needs to be something that you encounter every day.

It may also be helpful to choose a more durable cue that will remain consistent wherever you go. For example, if you know that you'll be brushing your teeth every day in the morning regardless of whether you're in Hong Kong or California, this would be a durable cue because you'll encounter it as a reminder to practice self-compassionate touch regardless of whether you're traveling or at home.

The cue should also occur in a location or environment that helps make the self-compassionate touch easier to do. In the same way you might find it helpful to study in a library or work out in the gym, many people find that self-compassionate touch is easier when done in private. For this reason, it may be helpful to choose a private space as your cue to practice self-compassionate touch, like a bathroom or your bedroom. The cue to the self-compassion practice should also be something that grabs your attention. For example, if your cue is at 9 in the morning, it may be especially helpful to set an alarm for this time. The best time to practice is often when you need it most in your day. Think about it. When in your day do you most need self-compassionate touch? When you're getting into bed and typically experiencing racing

thoughts? When you're getting dressed in the morning and worrying about your day? Walking into a space where you usually feel lonely or like you don't belong?

Also, it's important to be as specific as you can about what your cues look like. For example, if your cue is brushing your teeth, where are you brushing your teeth? What times of day are you brushing your teeth? Notice in the example here that the cue is three elements. The first is "after brushing my teeth." The second is "in the bathroom," and the third is "in the morning." The more elements you're able to use to describe your cue, the more specific your cue will be, and the more likely you'll be to practice self-compassionate touch. And especially in the first couple of weeks, if you notice that your cue is not working at all for you, feel free to change it. However, you don't want to change your cue too often, or you won't have the stability needed to develop your practice into a habit.

Rewards help to strengthen habit formation, and they strengthen our relationship to the cues that we choose. They're also closely linked to how you feel. A behavior that brings on positive or tones down negative emotions will be rewarding. So, the more you generate positive emotions like self-compassion and warmth towards yourself or tone down negative emotions like shame, sadness, or self-doubt, each time you practice self-compassionate touch, the easier it will be for you to form the practice into a habit.

Practice may also be important for experiencing the rewards of self-compassionate touch. For example, for most people, it's completely normal to not notice any benefits from self-compassionate touch right away. It may actually take a couple of weeks of daily practice before you start to notice yourself feeling more compassionate towards yourself—both while practicing and in your daily life. And the more you practice, the more effective it will be. You're not going to notice changes overnight, and you're not going to notice changes if you try it three times here

and there. Regular practice in the same context is key. Our suggestion is to have low expectations of noticing any differences within the first couple of weeks. This is when you're sowing the seeds of your practice. If you tend to practice regularly, you'll start to notice results. Finally, if you notice that you forgot to practice, celebrate this moment. You remembered to practice, and this is another chance to practice self-compassion towards yourself. Remember, when you really can't squeeze it in, feel free to give yourself up to two emergency skip days per week. In the same way that you can come back to school after a summer break and not forget everything you've learned, all practice adds up, regardless of how far apart. So, a caring friend to yourself might say that there's no need to beat up on yourself. Just pick up where you left off.

So, to summarize, the three most important ingredients for forming new habits are performing the behavior in response to the same cues, fully experiencing and savoring the rewards of that behavior, and practice, practice, practice! If you follow these steps, you'll be well on your way to forming this new habit. Next, using these ingredients, we're going to fill out a plan for building your self-compassionate touch habit. Click on the arrow below to continue.

References

1. Susman, E. S., Chen, S., Kring, A. M. & Harvey, A. G. Daily micropractice can augment single-session interventions: A randomized controlled trial of self-compassionate touch and examining their associations with habit formation in US college students. *Behaviour Research and Therapy* **175**, 104498 (2024).
2. Dreisoerner, A. *et al.* Self-soothing touch and being hugged reduce cortisol responses to stress: A randomized controlled trial on stress, physical touch, and social identity. *Comprehensive Psychoneuroendocrinology* **8**, 100091 (2021).
3. Neff, K. D. The criticizer, the criticized, and the compassionate observer. *Self-Compassion* https://self-compassion.org/exercise-4-supportive-touch/ (2015).
